# Power-scalable subcycle pulses from laser filaments

**DOI:** 10.1038/srep36263

**Published:** 2017-04-03

**Authors:** A.A. Voronin, A.M. Zheltikov

**Affiliations:** 1 Physics Department, International Laser Center, M. V. Lomonosov Moscow State University, Moscow 119992, Russia; 2 Department of Physics and Astronomy, Texas A&M University, 77843 College Station TX, USA; 3 Russian Quantum Center, 143025 Skolkovo, Moscow Region, Russia; 4 Kazan Quantum Center, A.N. Tupolev Kazan National Research Technical University, Chetaev 18a, 420126 Kazan, Russia

## Abstract

Compression of optical pulses to ultrashort pulse widths using methods of nonlinear optics is a well-established technology of modern laser science. Extending these methods to pulses with high peak powers, which become available due to the rapid progress of laser technologies, is, however, limited by the universal physical principles. With the ratio *P*/*P*_cr_ of the peak power of an ultrashort laser pulse, *P*, to the critical power of self-focusing, *P*_cr_, playing the role of the fundamental number-of-particles integral of motion of the nonlinear Schrödinger equation, keeping this ratio constant is a key principle for the power scaling of laser-induced filamentation. Here, we show, however, that, despite all the complexity of the underlying nonlinear physics, filamentation-assisted self-compression of ultrashort laser pulses in the regime of anomalous dispersion can be scaled within a broad range of peak powers against the principle of constant *P*/*P*_cr_. We identify filamentation self-compression scaling strategies whereby subcycle field waveforms with almost constant pulse widths can be generated without a dramatic degradation of beam quality within a broad range of peak powers, varying from just a few to hundreds of *P*_cr_.

Methods of pulse compression are of key significance for ultrafast optical science, playing a central role in the rapid progress of laser technologies toward unprecedentedly short pulse widths and extremely high peak powers^1^,^2^. At the forefront of this burgeoning field of research, technologies enabling the generation of electromagnetic lightwaves with temporal envelopes shorter than the field cycle have been developed^3^,^4^,^5^,^6^,^7^, offering unique tools for time-resolved studies of the fundamental physics behind light–matter interactions and paving the ways toward an ultimate time resolution in electron-dynamic studies and subcycle precision in lightwave sculpting. However, for high-peak-power ultrashort laser pulses, which become available due to rapidly progressing laser technologies, practical pulse-compression options are usually not many, if any at all, as laser damage severely limits conventional solid-state pulse-compression solutions.

Fortunately, laser-induced filamentation offers physical scenarios whereby high-peak-power ultrashort laser pulses can be compressed to few-cycle pulse widths^8^,^9^,^10^,^11^,^12^. With the advent of efficient and convenient parametric sources of ultrashort pulses in the mid-infrared^13^,^14^,^15^,^16^,^17^,^18^,^19^,^20^, the range where many solid-state materials exhibit anomalous dispersion, interesting and practically promising scenarios of filamentation-assisted pulse compression in the regime of anomalous dispersion have been realized^21^,^22^,^23^,^24^,^25^,^26^, opening the routes toward compact all-solid-state sources of few-cycle^21^,^22^, single-cycle^27^, and even subcycle^28^ pulses in the mid-infrared. If scaled from pulse energies of a few microjoules to tens of millijoules, these methods of pulse compression would ideally address the needs of rapidly developing technologies of short-pulse generation in the mid-infrared^20^,^29^.

Such a scaling, however, is nontrivial, and its very possibility is not obvious. While the key parameters of the most practically significant scenarios of laser-induced filamentation are known to obey, to a reasonable approximation, a set of physically transparent scaling laws in their behavior as functions of laser pulse widths, propagation paths, and beam-focusing geometries^30^,^31^,^32^, any scaling with respect to the ratio *P*/*P*_cr_ of the laser peak power, *P*, to the critical power of self-focusing, *P*_cr_, is inherently limited as this ratio controls the number of filaments that a high-*P* laser beam breaks up into and defines the limits of the single-filamentation regime^30^,^31^,^32^,^33^. Keeping this ratio constant is therefore one of the key principles in power scaling of laser filaments.

To make matters even more complicated, spatial modulation instabilities (MIs), which tend to build up in a laser beam with *P* well above *P*_cr_^34^, give rise to field hot spots across the beam^30^,^31^,^35^, increasing the risk of laser damage of any solid-state material, needed to provide an anomalous dispersion. Moreover, as a result of spatial MIs, which, in the case of sufficiently high field intensities, act jointly with ultrafast field-induced ionization, a laser beam breaks into multiple filaments, losing its quality and, eventually, its spatial coherence. With all these factors being a part of the picture, the question arises as to whether any reasonably power-scalable pulse compression scenario is possible in this regime.

Here, we show that, despite all the complexity of the underlying nonlinear physics, filamentation-assisted self-compression of ultrashort laser pulses in the regime of anomalous dispersion can be scaled within a broad range of peak powers against the principle of constant *P*/*P*_cr_. Based on the results of (3 + 1)-dimensional supercomputations, we identify filamentation self-compression scaling strategies whereby subcycle field waveforms with almost constant pulse widths can be generated without a dramatic degradation of beam quality within a broad range of *P*, varying from just a few to hundreds of *P*_cr_, enabling the generation of subcycle field waveforms with energies from tens of microjoules to several millijoules.

## General concept: Soliton self-compression

In one-dimensional pulse evolution, i.e., when the field in an optical waveform is allowed to be a function of only one, longitudinal spatial coordinate, while the dependence on the radial coordinates is suppressed, temporal self-compression is a universal behavior of ultrashort light pulses in the regime of anomalous dispersion^36^. This effect is widely used for pulse compression in optical fibers^37^ and is described by the canonical version of the nonlinear Schrödinger equation (NSE)^36^,^37^. As a part of their 1D soliton dynamics, ultrashort light pulses propagating in an anomalously dispersive medium with an instantaneous cubic nonlinearity with no high-order dispersion are known to display well-resolved cycles^37^, in which the phase of pulse compression is followed by pulse stretching. This oscillatory, breathing dynamics is fully controlled by the soliton number *N* = (*l*_d_/*l*_nl_)^1/2^, where 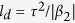 *l*_d_ is the dispersion length, *l*_nl_ = *λ*(2*πn*_2_*I*)^–1^ is the nonlinear length, *I* is the field intensity, *n*_2_ is the nonlinear refractive index, *τ* is the pulse width, *β*_2_ is the group-velocity dispersion coefficient, and *λ* is the wavelength. In this approximation, the minimum pulse width of a breathing soliton is achieved at the length *l*_*N*_ ≈ *πl*_d_/2(0.32/*N* + 1.1/*N*^2^)^37^.

Because of high-order dispersion, pulse self-steepening, and the inertial part of optical nonlinearity, the field waveform dynamics can substantially differ from this textbook scenario already in the 1D case. These differences become especially drastic if the pulse width achieved as a part of pulse self-compression dynamics becomes close to the field cycle^38^. Field evolution becomes even more complicated when the laser peak power *P* becomes higher than the critical power of self-focusing, *P*_cr_ = *Cλ*^2^/(8π*n*_0_*n*_2_), where *λ* is the wavelength, *n*_0_ is the field-free refractive index, *n*_2_ is the nonlinear refractive index, and *C* is a beam-profile-sensitive constant (for a Townes mode, *C* ≈ 3.72). In this regime, the field evolution in the time domain is strongly coupled to complex beam dynamics, which, in its turn, may drastically vary from the leading to the trailing edge of the pulse. Finally, in the *P* ≫ *P*_cr_ regime, any 1D treatment is expected to fail as the beam becomes inherently unstable with respect to spatial modulation instabilities, producing hot spots, breaking up into multiple filaments, eventually losing its connectedness and spatial coherence. Still, within a limited parameter space, clear signatures of solitonic pulse self-compression can be isolated even in this extreme regime of field evolution, enabling the generation of extremely short, subcycle optical field waveforms^39^. The main goal of our analysis here is to examine whether the power of these subcycle field waveforms can be scaled in the *P* ≫ *P*_cr_ regime of laser filamentation without dramatic degradation of beam quality.

One of the main difficulties of such a strategy when applied to a solid-state material is that, for such a material, there is no easy way to vary the critical power of self-focusing *P*_cr_, e.g., by changing the density of a nonlinear medium, as it would be possible in the gas phase. Thus, varying the laser peak power *P* will change the *P*/*P*_cr_ ratio as well. The strategy we are after should therefore enable power scaling of self-compression in a laser filament in violation of the principle of constant *P*/*P*_cr_. Such an approach can, of course, work only to a certain approximation, allowing, at best, a certain parameter, e.g, the pulse width *τ*_с_ at the point of maximum pulse compression, as in our approach, to be kept approximately constant within a reasonably broad range of *P*. While the key principles of this power scaling are based on qualitative physical arguments, including those borrowed from the 1D analysis, some elements of blind optimization within a limited parameter space will be inevitably needed to finely tune parameters for the most accurate power scaling of pulse self-compression in a laser filament.

## Modeling

Our numerical analysis is based on the three-dimensional time-dependent generalized nonlinear Schrödinger equation^30^,^31^ for the amplitude of the field, which is referred to hereinafter as the (3 + 1)-d GNSE model. This generalization of the nonlinear Schrödinger equation (NSE) includes all the key physical phenomena that have been identified as significant factors behind the spatiotemporal evolution of ultrashort optical pulses in nonlinear media, such as dispersion and absorption of the medium, beam diffraction, space-time focusing, Kerr nonlinearities, pulse self-steepening, spatial self-action phenomena, as well as ionization-induced loss, dispersion, and optical nonlinearities. In this model, the field evolution equation is solved jointly with the rate equation for the electron density *ρ(t*), which includes impact ionization and photoionization with the photoionization rate calculated using the Keldysh formalism^40^. Simulations are performed for typical parameters of YAG. The band gap is estimated as the band-edge value for YAG^41^, *E*_g_ ≈ 6.4 eV, although no significant changes were observed in simulations when the direct band-gap value of YAG^41^, *E’*_g_ ≈ 6.5 eV, was used. The Kerr-effect nonlinear refractive index is taken as *n*_2_ = 4 × 10^−16^ cm^2^/W to provide the best fit between simulations and experiments^42^,^43^,^44^. This value is within a factor of 1.75 of the *n*_2_ coefficient used in some of the earlier studies (e.g., in ref. 45), which is acceptable for a phenomenologically defined constant. Agreement between simulations and experiments^42^,^43^,^44^ also dictates a higher order Kerr effect (HOKE) coefficient *n*_4_ ≈ −1 × 10^−29 ^cm^4^/W^2^. This HOKE term is, in fact, one of the key limiting factors for the minimum pulse widths achieved in our simulations – compression to much shorter pulse widths becomes possible with *n*_4_ = 0.

Dispersion of YAG crystal was included in the model through a Sellmeier relation^46^. The zero group-velocity dispersion wavelength for YAG is *λ*_z_ ≈ 1610 nm. Similar to many other suitable materials, YAG exhibits anomalous dispersion in the long-wavelength part of the near-IR and in the mid-IR range. We therefore choose to work with an input field at a central wavelength *λ* = 3.9 μm. Sub-100-fs pulses with peak powers orders of magnitude higher than the self-focusing threshold for YAG (*P*_cr_ ≈ 30 MW at *λ* = 4 μm) can be delivered at this central wavelength by mid-infrared OPCPA sources^13^,^20^,^29^,^47^. Spatial modulation instabilities leading to the formation of multiple filaments are seeded in our model by superimposing a Gaussian-noise modulation on the input beam profile with a standard deviation of 4%^35^. Simulations were performed using an MPI parallel programming interface and the CUDA graphical architecture on the Lomonosov supercomputer cluster of Moscow State University.

## Soliton self-compression in 3D dynamics

**Figure 1:**
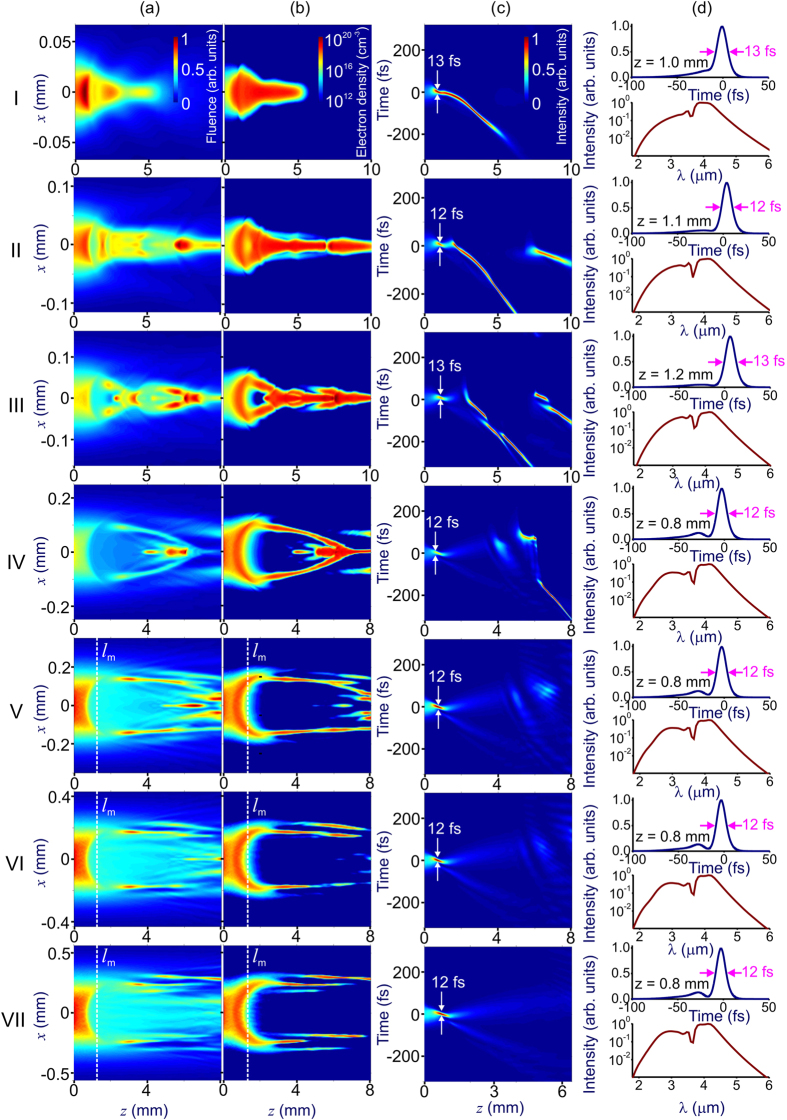
Spatiotemporal (3 + 1)-dimensional dynamics of a laser pulse with the central wavelength *λ*_0_ = 3.9 μm and the initial pulse width *τ*_0_ = 80 fs in an anomalously dispersive nonlinear medium: (**a**) the radial distribution of the field intensity integrated over the pulse as a function of the propagation coordinate, (**b**) electron density in the wake of the pulse, (**c**) spatiotemporal evolution of the field intensity, and (**d**) temporal envelope (top) and the spectrum (bottom) of the pulse on the beam axis at the point of maximum pulse compression. The peak power, *P*, energy, *W*, and beam diameter, *d*, of the input laser pulse are *P* = 5*P*_cr_, *W* = 13 μJ, *d* = 70 μm (row I), *P* = 15*P*_cr_, *W* = 40 μJ, *d* = 120 μm (row II), *P* = 30*P*_cr_, *W* = 75 μJ, *d* = 170 μm (row III), *P* = 100*P*_cr_, *W* = 0.25 mJ, *d* = 260 μm (row IV), *P* = 200*P*_cr_, *W* = 0.5 mJ, *d* = 370 μm (row V), *P* = 300*P*_cr_, *W* = 0.75 mJ, *d* = 450 μm (row VI), and *P* = 500*P*_cr_, *W* = 1.25 mJ, *d* = 590 μm (row VII).

**Figure 2:**
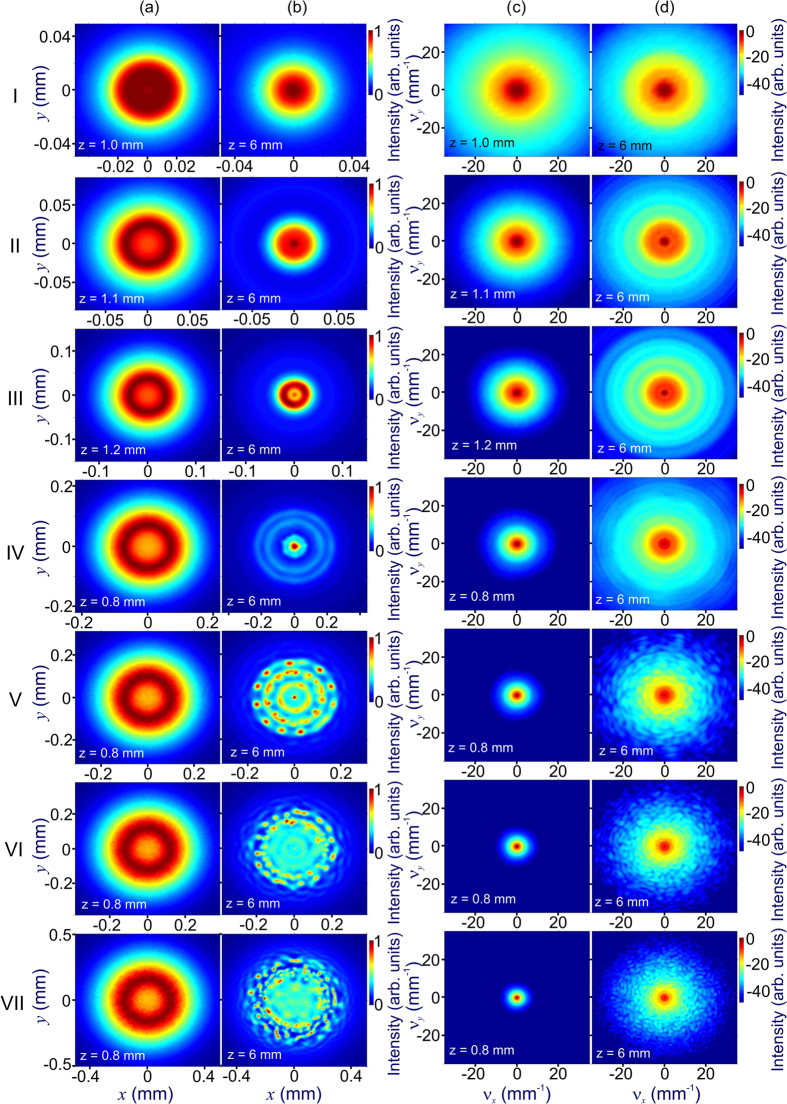
Multiple filamentation dynamics of a laser pulse with the central wavelength *λ*_0_ = 3.9 μm and the initial pulse width *τ*_0_ = 80 fs in an anomalously dispersive nonlinear medium: (**a**,**b**) transverse beam profiles and (**c**,**d**) the angular spectra at the point of maximum pulse compression (**a**,**c**) and at z = 6 mm (**b**,**d**). Parameters of the input pulse in rows I–VIII are as specified in Fig. 1.

Results of (3 + 1)-d GNSE simulations presented in Figs 1 and 2 illustrate the key tendencies of filamentation dynamics of ultrashort laser pulses in an anomalously dispersive medium. In the time domain (Fig. 1c), the pulse is seen to undergo self-compression at the first stage of its spatiotemporal evolution, reaching, for suitably chosen input parameters (Fig. 1c), subcycle pulse widths at the point of maximum pulse compression, *z* ≈ *l*_*c*_. This pulse compression stage is followed by post-compression pulse stretching, accompanied, in the *P* ≫ *P*_cr_ regime, by beam break up into multiple filaments (to the right of the vertical dashed lines in rows V–VII in Fig. 1a,b).

**Figure 3:**
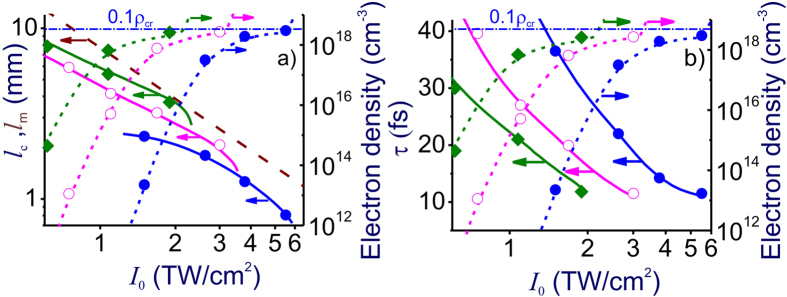
The length of maximum pulse compression ((**a**) left axis) and the pulse width *τ*_c_ at the point of maximum pulse compression ((**b**) left axis) as functions of the input field intensity calculated using the (3 + 1)-dimensional model (filled circles, open circles, and diamonds) and the 1D GNSE (solid lines). The right axes show the maximum electron density *ρ*_m_ within a filament calculated as a function of the driver intensity using the (3 + 1)-dimensional model (filled circles, open circles, and diamonds) and the 1D GNSE (dotted lines). Also shown (dashed line) is the MI buildup length *l*_m_ as predicted by the Bespalov—Talanov theory. The horizontal dash—dotted line shows the 0.1*ρ*_cr_ level of electron density, taken in our analysis as a criterion of increased risk of laser damage of the material. The input pulse width is *τ*_0_ = 80 fs (blue line and filled circles), 150 fs (red line and open circles), and 250 fs (green line and diamonds). The central wavelength of the laser pulse is *λ*_0_ = 3.9 μm.

Although pulse self-compression dynamics in the regime of anomalous dispersion has been thoroughly understood for optical fibers, in the case of freely propagating beams, this self-compression behavior, as can be seen in Figs 1 and 2, is observed as a part of complex spatiotemporal field evolution, involving diffraction, self-focusing due to the Kerr nonlinearity, defocusing and scattering by the transverse profile of the electron density induced by ultrafast photoionization, as well as beam filamentation, spatial modulation instabilities, ionization-induced blue shift, shock waves, and the high-order Kerr effect. Results of simulations presented in rows V–VII of Fig. 2b,d show that, in the regime of *P* ≫ *P*_cr_, not only the one-dimensional analysis, but any treatment assuming that the beam preserves its radial symmetry fails as the beam becomes intrinsically unstable with respect to spatial modulation instabilities (rows V–VII in Fig. 2b,d), amplifying random field-intensity fluctuations, which feature no symmetry whatsoever. These instabilities give rise to hot spots randomly distributed across the laser beam (rows V–VII in Fig. 2b), induce random, asymmetric sidelobes in the angular spectra (rows V–VII in Fig. 2d), and lead to beam breakup into multiple filaments (rows V–VII in Figs 1a and 2b), increasing the risk of optical damage and dramatically lowering the output beam quality. The input parameters in simulations presented in Figs 1 and 2 are chosen in such a way as to keep the electron density *ρ* at any point of a filament below 0.1*ρ*_cr_ (shown by the horizontal dash–dotted line in Fig. 3a,b), *ρ*_cr_ being the critical plasma density for given *λ*. However, with plasma refraction enhanced for longer wavelengths, excessive ionization can also give rise to unwanted beam scattering, which tends to grow toward the trailing edge of the pulse, giving rise to conical patterns in output beam profiles (rows II–VII in Fig. 2a), thus also lowering the beam quality of compressed pulses.

Simulations presented in Figs 1 and 2 are instructive in showing that a finite length *l*_m_ is needed for modulation instabilities to build up across a laser beam from the noise level, giving rise to multiple filaments at later stages of field evolution. With input beam parameters in all simulations presented in Figs 1 and 2 chosen in such a way as to keep the diffraction length *l*_df_ = *kw*_0_^2^ (*k* = 2*πn*_0_*/λ* and *w*_0_ is the beam radius) long compared to *l*_m_, a simple estimate *l*_m_ ≈ 5*l*_nl_, which corresponds to an MI gain of exp(5) according to the Bespalov–Talanov (BT) MI theory^34^, agrees reasonably well with the MI buildup length in (3 + 1)-d simulations. Moreover, as long as the soliton compression length *l*_c_ is kept shorter than both *l*_df_ and *l*_m_, both the length at which the minimum pulse width is achieved in (3 + 1)-d simulations in Fig. 1c and the minimum pulse width *τ*_c_ in these simulations are in almost perfect agreement (Fig. 3a,b) with predictions of the 1D GNSE model^37^ – one-dimensional generalization of the NSE, which includes high-order dispersion, inertia and dispersion of optical nonlinearity, and field-induced ionization^30^,^31^.

Based on these observations, we use the results of 1D analysis and predictions of the BT theory as a reference and a guide to define, in a rough approximation, a limited area within the parameter space within which *P*-scalable subcycle pulse generation with approximately constant pulse width *τ*_с_ at the point of maximum pulse compression can be expected in laser filaments. Full (3 + 1)-dimensional simulations are then performed to finely tune parameters within this area, often via a blind search, for the best *P* scalability of this regime of subcycle pulse generation.

To include limitations related to the laser damage in our model, we use a standard quantitative measure for the laser breakdown threshold^48^,^49^, loosely defined as *ρ*_th_ ≈ 0.1*ρ*_cr_, with *ρ*_cr_ being the critical plasma density for given *λ*, and require that the global maximum electron density, *ρ*_m_, achieved at any point inside the material at any instant of time be less than 0.1*ρ*_cr_. For relatively low laser peak powers, the global maximum of the electron density is typically observed on the beam axis (Fig. 1a). In the high-*P* regime, however, this maximum can be achieved at one of the MI-induced field hot spots, randomly distributed over the laser beam (Fig. 2b,d). In Fig. 3a,b, we show *ρ*_m_ calculated as a function of the field intensity *I* for three different input pulse widths. The limiting admissible laser intensities and, hence, the input peak powers are found from the points where the *ρ*_m_(*I*) dependences cross the *ρ*_th_ = 0.1*ρ*_cr_ line (dashed horizontal lines in Fig. 3a,b). In particular, for an input pulse width *τ*_0_ = 80 fs, that is, the *τ*_0_ value used in simulations presented in Figs 1 and 2, laser-damage considerations dictate a limiting admissible input intensity of about 5.8 TW/cm^2^.

## Power-scalable subcycle pulses

**Figure 4:**
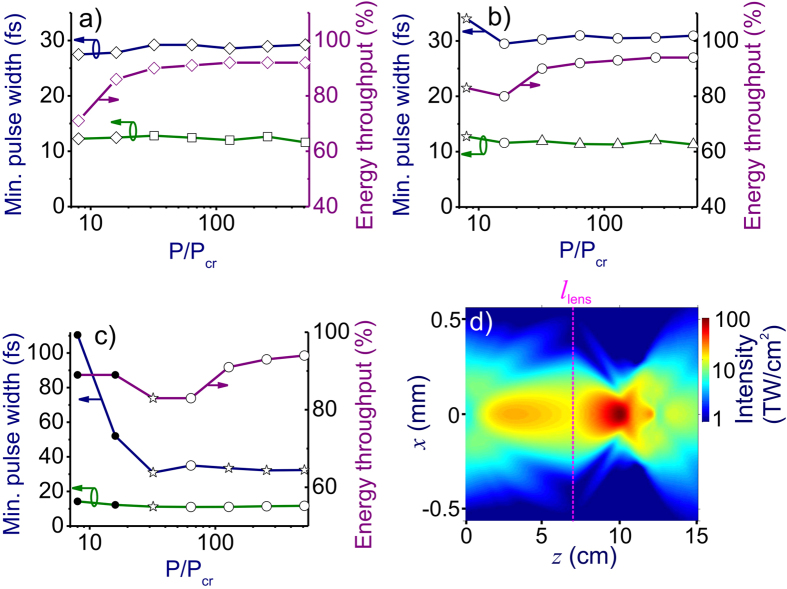
(**a**–**c**) The minimum on-axis pulse width *τ*_c_ (green), the minimum beam-integrated pulse width *τ*’_с_ (blue), and the whole-beam energy throughput *ξ* (purple) calculated using the (3 + 1)-dimensional model as functions of the *P*/*P*_cr_ ratio. The input pulse width is *τ*_0_ = 80 fs (**a**), 150 fs (**b**), and 250 fs (**c**). The input field intensity is 1.0 TW/cm^2^ (filled circles), 1.5 TW/cm^2^ (asterisks), 2.0 TW/cm^2^ (open circles), 3.0 TW/cm^2^ (triangles), 4.0 TW/cm^2^ (diamonds), and 5.5 TW/cm^2^ (squares). The central wavelength of the laser pulse is *λ*_0_ = 3.9 μm. (**d**) Two-dimensional map of the beam profile evolution in a vacuum behind a YAG plate with a thickness set exactly equal to *l*_c_ with a 3-cm-focal-length lens or mirror placed at a distance of *l*_lens_ ≈ 7 mm from the exit surface (shown by the vertical dashed line). The input peak power is 500 *P*_cr_. The input pulse width is 80 fs.

Power scalability of subcycle waveform generation through filamentation-assisted pulse self-compression is illustrated in Fig. 4a–c, which present the pulse width at the point of maximum pulse compression calculated as a function of the *P*/*P*_cr_ ratio. Each point in these plots represents a full (3 + 1)-d simulation for an individual set of initial parameters (specified in the figure caption) chosen in such a way as to meet the conditions *l*_c_ < *l*_df_, *l*_m_ and *ρ*_m_ < 0.1*ρ*_cr_. The lower curves show the minimum pulse width *τ*_с_ on the beam axis, while the upper curves display the pulse width *τ′*_с_ of the temporal envelope or the laser power integrated over the entire beam, 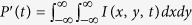. The optimal sets of parameters needed to achieve the most efficient pulse compression on the beam axis and within the entire beam, as well as the propagation paths needed to achieve these minima, as can be seen from Fig. 4a–c, are generally different.

Due to the remarkably efficient pulse self-compression within the entire beam (the upper curves in Fig. 4a–c), no beam diaphragming with a finite-diameter pinhole is necessary for a high energy throughput of pulse compression. The energy throughput for such whole-beam pulse self-compression, defined as the ratio *ξ* = *W*_c_/*W*_0_ of the energy *W*_c_ within the entire beam at the point of maximum pulse compression, *z* = *l*_c_, to the input pulse energy *W*_0_ is also presented in Fig. 4a–c, showing that whole-beam self-compression to sub-30-fs pulse widths with an energy throughput above 90% is possible within a broad range of *P*/*P*_cr_ ratios (e.g., for *P*/*P*_cr_ ranging from 30 to 500 in Fig. 4a,b and *P*/*P*_cr_ from 100 to 500 in Fig. 4c).

With the conditions *l*_c_ < *l*_df_, *l*_m_ and *ρ*_m_ < 0.1*ρ*_cr_ satisfied for all the calculations presented in Figs 1 and 2, the adverse effect of MIs and multiple filamentation on the beam quality at *z* = *l*_c_ is minimized. This is verified by the transverse beam profiles at the point of maximum pulse compression and their angular spectra presented in Fig. 2a. However, as the optical field propagates further on along the *z*-axis, well-resolved signatures of growing MIs show up in both transverse beam profiles (rows V–VII in Fig. 2b) and angular spectra (rows V–VII in Fig. 2d), indicating a rapid degradation of beam quality.

As the most striking result, simulations presented in Figs 1c,d and 3a–c show that subcycle pulse widths *τ*_с_ can be maintained within a broad range of laser peak powers, varying from just a few to hundreds of critical powers of self-focusing. As can be seen from these simulations, optical waveforms with a minimum pulse width of 12–13 fs can be generated for laser peak powers from, roughly, 5*P*_cr_ up to at least 500*P*_cr_. The main physical factors that limit power scalability of subcycle waveforms to even higher *P* include the increased risk of optical damage, the exponentially growing gain of MIs, excessive plasma scattering, as well as pulse breakup before the point of maximum pulse compression through the fission of soliton transients enhanced by ultrafast ionization^38^.

As a typical behavior of their temporal envelope, optical waveforms produced at the point of maximum pulse compression feature an intense subcycle peak with a pulse width *τ*_с_ ≈ 12–13 fs, which is shifted toward the back of the pulse (Fig. 1d). This time shift is due to optical shock-wave effects which, similar to 1D pulse compression scenarios in gas-filled hollow waveguides^38^, play a significant role in subcycle pulse generation in laser filaments. In the regime considered here, shock-wave effects enhance the spectral broadening of the high-frequency part of the spectrum and steepen the trailing edge of the field waveform (Fig. 1c,d). With this waveform steepening, the energy is no longer uniformly distributed over multiple individual pulses forming a breathing soliton, but is concentrated within one of the soliton transients toward the back of the pulse. This effect, acting jointly with ionization-induced enhancement of the high-frequency part of the spectrum (Fig. 1d), assists a synthesis of an isolated subcycle soliton feature shifted toward the trailing edge of the pulse, as shown in Fig. 1d.

At the carrier wavelength *λ* = 3.9 μm, the pulse width *τ*_с_ ≈ 12–13 fs of the most intense peak in the compressed pulse envelope (Fig. 1d) corresponds to 0.9 field cycles. Being shifted toward the back of the original laser pulse, this subcycle field transient is preceded by a pedestal, which contains 20–23% of the total energy of the compressed pulse and whose peak intensity is 10–15 times lower than the intensity at the center of the 12–13-fs main peak. For the input peak power *P* = 500*P*_cr_, input energy *W* = 1.25 mJ, and beam diameter of the input pulse *d* = 590 μm (row VII in Figs 1 and 2), the 12-fs peak of the compressed pulse has an energy of 0.4 mJ, translating into a peak power of 35 GW, which is 2.1 times higher than the input peak power. With the thickness of the nonlinear medium set exactly equal to *l*_c_, a 3-cm-focal-length lens or mirror placed at a distance of 7 cm from the exit surface will focus this beam into a spot with a diameter of about 140 μm, giving rise to a field intensity of 100 TW/cm^2^ at the focal plane (Fig. 4e).

Unlike (3 + 1)-d simulations, which provide a quantitative understanding of hot-spot formation and multiple filamentation across a laser beam in the *P*/*P*_cr_ ≫ 1 regime (Figs 1 and 2), (2 + 1)-d models can at best only offer qualitative insights, but cannot quantitatively describe high-*P* beam breakup and multiple filamentation, as they rely on the assumption that the beam preserves its axial symmetry, which fails for *P*/*P*_cr_ ≫ 1. When dealing with the beam breakup due to MIs and multiple filamentation, (2 + 1)-d studies have to rely on simple qualitative criteria, which can be formulated, e.g., in terms of quite arbitrary limitations on the *B* integral (e.g., ref. 50). Such an approach can help define the parameter space where MIs and multiple filamentation can be avoided, but cannot help examine the influence of MIs and multiple filamentation on pulse evolution. This explains why the use of much more powerful computer resources (supercomputer simulations are usually necessary for (3 + 1)-d analysis, while (2 + 1)-d simulations can be run on a personal computer) is indeed justified whenever quantitative picture of multiple filamentation is needed, e.g., when the results of simulations have to be used for a predictive modeling of filamentation self-compression experiments^42^,^43^,^44^.

We emphasize that the scaling of filamentation-assisted self-compression of high-*P* laser pulses identified in this work is more general than and is not reduced to the scaling of pulse self-compression based on the intensity dependence of small-scale self-focusing (e.g., ref. 50). While the latter approach suggests that the peak power of the pulse can be scaled by simply keeping its intensity constant, the approach advocated here is not reduced to a limiting case of large Marburger self-focusing lengths, but also extends the scaling of filamentation pulse self-compression to the case when the Marburger self-focusing length *l*_M_ is comparable with the pulse self-compression length *l*_c_, so that the beam undergoes self-focusing as a whole. In particular, in lines I, II, and III in Fig. 1, the self compression length *l*_c_ is estimated as 1.0, 1.1, and 1.2 mm, respectively, while the Marburger length *l*_M_ is 0.95, 1.3, and 1.7 mm, respectively. As a result, the beam dynamics, as can be seen from the maps presented in lines I, II, and III in Fig. 1 is dominated by the self-focusing of the beam as a whole.

## 3D soliton self-compression in laser filaments versus light bullets

**Figure 5:**
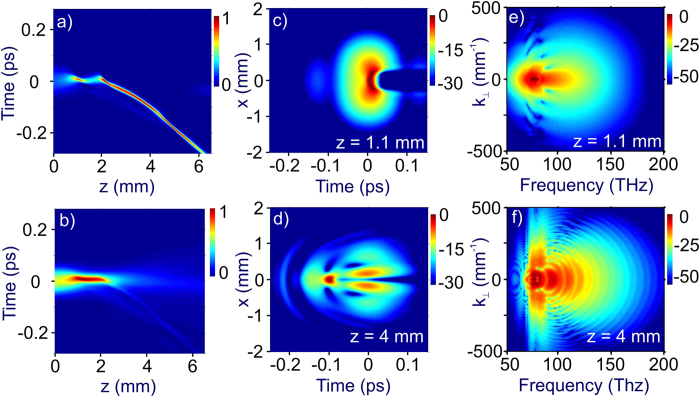
Spatiotemporal (3 + 1)-dimensional dynamics of a laser pulse with *λ*_0_ = 3.9 μm, *τ*_0_ = 80 fs, *P* = 15*P*_cr_, *W* = 40 μJ, and *d* = 120 μm: (**a**,**b**) spatiotemporal evolution of (**a**) the on-axis field intensity and (**b**) field intensity integrated over the beam, (**c**,**d**) the maps of the field intensity resolved in time and the radial coordinate *x* at *z* = 1.1 mm (**c**) and *z* = 4 mm (**d**), (**e**,**f**) the angular spectra of the pulse at *z* = 1.1 mm (**e**) and *z* = 4 mm (**f**).

Simulations presented in Fig. 1c demonstrate that the filamentation-assisted soliton pulse self-compression considered in the previous sections of this paper can take place in the regimes where the generation of light bullets^51^,^52^,^53^ is also possible (Fig. 1c, rows I–III). Generally, even when light bullet formation and filamentation-assisted soliton self-compression occur in the same parameter space (Fig. 1c, rows I–III), these phenomena are separated in space (Fig. 1c, rows II and III) unless the parameters are chosen in a special way, such that *l*_M_ ≈ *l*_c_. In this special case, the point of maximum soliton pulse compression can coincide with the point where light bullets are generated (Fig. 1c, row I). A distinctly different physics behind these two processes is illustrated in Fig. 5, which presents the details of spatiotemporal pulse evolution shown in row II of Figs 1 and 2. Here, the laser pulse is seen to undergo pulse self-compression, reaching a minimum on-axis pulse width of 12 fs at *z* ≈ *l*_c_ ≈ 1.1 mm (Fig. 5a). Formation of a light bullet, on the other hand, is not observed until *z* ≈ 2 mm (Fig. 5a,b). Starting with this point, typical features of a light bullet can be readily recognized. Unlike the self-compressed pulse at *z* ≈ *l*_c_ ≈ 1.1 mm (Fig. 5c), the light bullet is shifted along the time axis, with its maximum localized at *τ* ≈ −0.1 ps at *z* ≈ 4 mm (Fig. 5b,d), due to a characteristic phase matching in a light bullet^54^,^55^.

While light bullets need a reservoir of energy to support their propagation even in vacuum^26^, filament-compressed subcycle pulses studied in this work, once created, can propagate without any external energy supply. In particular, unlike light bullets, such pulses can be refocused and transmitted over large distances, of course, subject to the laws of dispersion and diffraction. As a result, the temporal properties of light bullets and subcycle pulses generated through soliton self-compression are very different. The pulse width in the light bullet on the beam axis is very short, about 12 fs at *z* ≈ 4 mm (Fig. 5a). However, because of the energy reservoir needed for the existence of a light bullet^26^,^56^, when integrated over the entire beam, the pulse width at *z* ≈ 4 mm is about 80 fs (Fig. 5b). For a self-compressed pulse at *z* ≈ *l*_c_ ≈ 1.1 mm, on the other hand, the beam-integrated pulse width is much shorter, about 28 fs (Fig. 5b), since this pulse does not require an external energy reservoir.

In simulations presented in rows II and III of Fig. 1c, the Marburger self focusing length, *l*_M_ ≈ 1.3 and 1.7 mm for rows II and III in Fig. 1, respectively, is larger than the soliton self-compression length, *l*_c_ ≈ 1.1 mm. As a result, after the first compression at *z* ≈ *l*_c_ ≈ 1.1 mm, the pulse undergoes a second cycle of compression at a distance *z* ≈ 1.8 mm in row II in Fig. 1c (also Fig. 5a) and *z* ≈ 2.9 mm in row III in Fig. 1c, which in both cases is slightly larger than *l*_M_ because of ionization-induced defocusing.

The difference in phase matching controlling the generation of new frequency components in the light bullet and in the self-compressed pulse at *z* ≈ *l*_c_ ≈ 1.1 mm is also clearly seen in the angular spectra presented in Fig. 5e,f. Specifically, signature circular fringes seen in Fig. 5f are typical of light bullets^57^,^58^, indicating efficient generation of new frequency components at large angles with respect to the beam axis. The angular spectrum of the compressed pulse at *z* ≈ *l*_c_ ≈ 1.1 mm, on the other hand, does not exhibit such circular fringes, as new frequency components are predominantly generated along the beam axis, explaining, in particular, why the insights obtained from 1D treatment are so helpful for the full 3D analysis of this pulse self-compression scenario.

While light-bullet formation is distinctly different from the filamentation-assisted pulse self-compression scenario examined in this work, dynamics of soliton self-compression is, on the opposite, very relevant, offering important physical insights into the considered scenario of subcycle pulse generation. Well-resolved soliton features can be readily identified in the considered filamentation pulse self-compression dynamics. Indeed, behind the point of maximum pulse self-compression (*z* ≈ 1.1 mm in row II of Fig. 1c and in Fig. 5a), the pulse undergoes a stretching phase again, following the evolution of a high-order soliton with a soliton number *N* ≈ 2.5. In particular, the length of maximum pulse compression in 1D dynamics of an *N* = 2.5 soliton, *l*_*N*_ ≈ 1.16 mm, agrees very well with the length of maximum pulse compression in 3D simulations, *l*_c_ ≈ 1.1 mm.

## Conclusion

To summarize, we have shown that, despite all the complexity of the underlying nonlinear physics, filamentation-assisted self-compression of ultrashort laser pulses in the regime of anomalous dispersion can be scaled within a broad range of peak powers against the principle of constant *P*/*P*_cr_. Based on the results of (3 + 1)-dimensional supercomputations, we have identified filamentation self-compression scaling strategies whereby subcycle field waveforms with almost constant pulse widths can be generated without a dramatic degradation of beam quality within a broad range of *P*, varying from just a few to hundreds of *P*_cr_, enabling the generation of subcycle field waveforms with energies from tens of microjoules to several millijoules.

## Additional Information

**How to cite this article**: Voronin, A.A. and Zheltikov, A.M. Power-scalable subcycle pulses from laser filaments. *Sci. Rep.* **6**, 36263; doi: 10.1038/srep36263 (2016).

**Publisher's note:** Springer Nature remains neutral with regard to jurisdictional claims in published maps and institutional affiliations.
